# Inflammatory myopathies and beyond: The dual role of neutrophils in muscle damage and regeneration

**DOI:** 10.3389/fimmu.2023.1113214

**Published:** 2023-02-27

**Authors:** Jiram Torres-Ruiz, Beatriz Alcalá-Carmona, Ricardo Alejandre-Aguilar, Diana Gómez-Martín

**Affiliations:** ^1^ Department of Immunology and Rheumatology, Instituto Nacional de Ciencias Médicas y Nutrición Salvador Zubirán, Mexico City, Mexico; ^2^ Laboratory of Entomology, Department of Parasitology, Escuela Nacional de Ciencias Biológicas, Instituto Politécnico Nacional, Mexico City, Mexico

**Keywords:** muscle regeneration, neutrophils, neutrophil extracellular traps, low density granulocytes, myositis

## Abstract

Skeletal muscle is one of the most abundant tissues of the human body and is responsible for the generation of movement. Muscle injuries can lead to severe disability. Skeletal muscle is characterized by an important regeneration capacity, which is possible due to the interaction between the myoblasts and immune cells. Neutrophils are fundamental as inducers of muscle damage and as promoters of the initial inflammatory response which eventually allows the muscle repair. The main functions of the neutrophils are phagocytosis, respiratory burst, degranulation, and the production of neutrophil extracellular traps (NETs). An overactivation of neutrophils after muscle injuries may lead to an expansion of the initial damage and can hamper the successful muscle repair. The importance of neutrophils as inducers of muscle damage extends beyond acute muscle injury and recently, neutrophils have become more relevant as part of the immunopathogenesis of chronic muscle diseases like idiopathic inflammatory myopathies (IIM). This heterogeneous group of systemic autoimmune diseases is characterized by the presence of muscle inflammation with a variable amount of extramuscular features. In IIM, neutrophils have been found to have a role as biomarkers of disease activity, and their expansion in peripheral blood is related to certain clinical features like interstitial lung disease (ILD) and cancer. On the other hand, low density granulocytes (LDG) are a distinctive subtype of neutrophils characterized by an enhanced production of NETs. These cells along with the NETs have also been related to disease activity and certain clinical features like ILD, vasculopathy, calcinosis, dermatosis, and cutaneous ulcers. The role of NETs in the immunopathogenesis of IIM is supported by an enhanced production and deficient degradation of NETs that have been observed in patients with dermatomyositis and anti-synthetase syndrome. Finally, new interest has arisen in the study of other phenotypes of LDG with a phenotype corresponding to myeloid-derived suppressor cells, which were also found to be expanded in patients with IIM and were related to disease activity. In this review, we discuss the role of neutrophils as both orchestrators of muscle repair and inducers of muscle damage, focusing on the immunopathogenesis of IIM.

## Introduction

Skeletal muscle is one of the most abundant tissues of the human body, representing 40-45% of its total mass and is responsible for the generation of movement (Reviewed in 1). Muscle injury can stem from a variety of events including muscle lacerations, contusions, strains and chronic diseases (inflammatory myopathies, muscular dystrophies and neuropathies) (1). A loss of over 20% of muscle volume (volumetric muscle loss) can be the result of orthopedic or oncological surgeries and critically hampers the muscle regenerative capacity (1).

Regarding sports-related damage, contusion and strain lesions represent approximately 90% of all exercise-related injuries (Reviewed in 2). Exercise-induced muscle damage encompasses a combination of transient myofibrillar disruption, loss of muscle strength and power, delayed onset muscle soreness, swelling, reduced range of motion, and the systemic efflux of myocellular enzymes and proteins (Reviewed in 3). Mechanical strain induces an overload of the sarcolemma and T-tubule structures, which leads to opening of stretch-activated channels, membrane disruption, and excitation-contraction coupling dysfunction (3). The intracellular calcium stimulates the calpain enzymes that degrade contractile proteins resulting in prolonged loss of muscle strength (3). Muscle injuries can lead to serious disability and therefore, a lot of effort has been taken to design muscle regeneration strategies (1).

Muscle regeneration is best studied in animal models, which according to Mahdy et al, consist in inducing chemical injuries (bupivacaine, cardiotoxin and glycerol injections), or physical lesions (freezing and crushing injuries, and surgical models like denervation and tenotomy) (Reviewed in 4). Skeletal muscle has a very important regeneration capacity which is dependent on the presence of myoblasts (muscle progenitor cells, MPC), that represent 5-10% of the muscle cells in adults (Reviewed in 5). Muscle repair requires an interaction between MPC, inflammatory, vascular, and stromal cells in the extracellular matrix (3). After a muscle injury, neutrophils are fundamental for the initiation of an inflammatory response, that is crucial for the regeneration and functional recovery of the muscle. On the other hand, neutrophils are important effectors of organ damage in a group of systemic autoimmune diseases called idiopathic inflammatory myopathies (IIM). Neutrophils have recently become more relevant as biomarkers of disease activity and as inducers of the clinical features of patients with dermatomyositis (DM and anti-synthetase syndrome (AS). In this review, we address the dual role of neutrophils as inducers of muscle damage and effectors in the regeneration phase after a muscle injury. Also, focusing on inflammatory myopathies, we describe the role of neutrophils as biomarkers and effectors of organ damage in this group of diseases.

## Effector functions of neutrophils

As part of the first line of defense against microorganisms, neutrophils possess many different effector functions which can also cause tissue damage. These functions are degranulation, phagocytosis, respiratory burst and the production of neutrophil extracellular traps (NETs) (Reviewed in 6). Phagocytosis is quick and is enhanced by complement system, IgG and cell priming (6). After opsonization, the Fcγ receptors, C-type lectins and complement receptors also favor the phagocytosis process (6). Once phagocytosis occurs, primary and secondary granules fuse with the phagosome, releasing antimicrobial enzymes. Also, there is a respiratory burst mediated by the NADPH (nicotinamide adenine dinucleotide phosphate) oxidase complex (6). Another enzyme responsible for the production of oxidants is myeloperoxidase (MPO), which produces hypochlorous acid (6). Neutrophils possess three types of granules, the primary or azurophils contain MPO, cathepsin G, neutrophil elastase (NE), protein 3 and defensins. The secondary granules contain lactoferrin and the tertiary contain gelatinase, and matrix metalloproteinase-9 (MMP9) (6). The secretory vesicles of the neutrophils contain albumin and preformed cytokines (6). The release of the granule content of the neutrophils (degranulation) occur in the following order: secretory vesicles, tertiary, secondary and primary granules (6).

Also, neutrophils are responsible for the production of extracellular traps (NETs). The NETs are web-like structures composed by chromatin decorated with the content of the granules of the neutrophils (6). The production of NETs can be triggered by microbes, including bacteria, fungi, protozoa, and viruses, severe infections, immune complexes, cytokines, chemokines (interleukin (IL)-8, tumor necrosis factor (TNF)-α) and microcrystals among others (Reviewed in 7). Therefore, the proinflammatory state observed in autoimmune disorders like systemic lupus erythematosus (SLE), rheumatoid arthritis (RA), and IIM promotes the production of NETs (Reviewed in 8)

The process to produce NETs initiates with the synthesis of reactive oxygen species and then, the PAD4 (peptidyl arginine deiminase 4) decondense the chromatin through the citrullination of histones. NE and MPO also contribute to the decondensation of chromatin. Afterwards, the nuclear and cell membranes are degraded and the NETs are released to the extracellular space (Reviewed in 9).

Currently, two main processes leading to the production of NETs have been described:

1. Suicidal NETosis: This is considered a special type of cell death that follows the mechanism of NETosis described above. There is chromatin decondensation with rupture of the cell membrane and cell death in a lapse of 2–4 h. It is highly pro-inflammatory and has been observed in most of NETs-induced damage in autoimmune diseases, such as IIM (7, 10).
2. Vital release of NETs: It is triggered by the stimulation of toll-like receptors (TLR) and the C3 receptor. Also, the release of mitochondrial DNA (mtDNA) without loss of viability has been observed after the stimulation of neutrophils with granulocyte macrophage colony stimulating factor (GM-CSF) and lipopolysaccharide (LPS) (7). The vital release of NETs occurs in a lapse of 5–60 min and it is a fundamental antimicrobial defense mechanism (9). The secretion of nuclear DNA is made by vesicular trafficking from the nucleus to the extracellular space (9). After this process, neutrophils maintain their phagocytic and chemotactic capacities (9).

Another relevant classification based on the mechanisms of the release of NETs is their dependence on the production of ROS by the NADPH oxidase (7). NETosis induced by *S. aureus* or phorbol 12-myristate 13-acetate (PMA) is dependent on NADPH oxidase (7). On the other hand, the release of NETs after stimulation with calcium ionophores is mediated by the production of mitochondrial ROS (7).

Albeit the main function of neutrophils is the defense against microbes, they are also fundamental in the tissue repair process. In the following section, we will discuss the role of neutrophils in the muscle regeneration after injury.

## Neutrophils as key players in muscle regeneration after injury

The successful muscle regeneration is the result of the balance between a group of sequential steps that include the phases of inflammation and degeneration, muscle regeneration and remodeling, which are discussed below. In this review, we will only assess the role of neutrophils on muscle regeneration after sterile injuries.

## Inflammation and degeneration

The first phase of the muscle repair after injury is the inflammation and degeneration of the muscle fibers with hematoma formation and necrosis of the myotubes. The necrosis of muscle fibers promotes the liberation of damage associated molecular patterns (DAMP) such as high mobility group box 1 (HMGB1), adenosine triphosphate (ATP), single stranded ribonucleic acid (ssRNA), hyaluronic acid and heat shock proteins (11). The liberation of DAMP signals from muscle cells is especially prominent in ischemia-reperfusion (IR) injuries. In this type of lesion, the severe hypoxia favors the anaerobic metabolism, promoting the accumulation of lactic acid with the consequent metabolic acidosis, mitochondrial dysfunction, oxidative stress, local inflammation and eventually tissue necrosis, leading to an intense liberation of DAMP and other intracellular components (12). After reperfusion, the DAMP stimulate the TLR expressed in neutrophils and macrophages (11), promoting a severe inflammatory response (12). If the inflammation is properly regulated, the outcome will likely be a complete muscle recovery (13) nonetheless, if an initially extensive damage is further enhanced by neutrophils, the IR injuries may lead to muscle fibrosis and loss of function due to an excessive inflammatory response (12). The liberation of DAMP is not the only mechanism responsible for the initial inflammatory response after muscle injury. MPC also contribute to the generation of inflammation by attracting monocytes through the expression of macrophage-derived chemokine (MDC), monocyte chemoattractant protein-1 (MCP-1), fractalkine, and vascular endothelial growth factor (VEGF) (Reviewed in 14).

## Role of neutrophils in the Initial inflammatory response after muscle injury

Within the first 24 hours after the lesion, neutrophils are attracted to the muscle by complement (C5), prostaglandins, leukotrienes, factors released by platelets (thromboxane A_2_, 5-hydroxitryptamine and histamine) (2) and by the secretion of CXCL1 (CXC motif chemokine ligand 1) and CCL2/MCP-1 (CC motif chemokine ligand 2 or monocyte chemoattractant protein-1) by resident macrophages (Reviewed in 15). As previously reviewed, neutrophils peak at 6-24 hours and rapidly decline 72-96 hours after the injury (Reviewed in 16). After their transmigration, neutrophils release NE, MPO and reactive species of oxygen and nitrogen (3). Also, according to the review by Toumi et al, neutrophils become activated in this phase, with the consequent induction of the respiratory burst and degranulation, which may amplify the initial muscle damage (Reviewed in 17). During the initial phase of the muscle injury, there is vascular lesion with low oxygen supply and decreased pH values (14). In this regard, the ROS from neutrophils contribute to the muscle fibers’ degradation and vascular lesions after IR injury (16).

The neutrophil invasion of the muscle is a generic and indispensable response to acute muscle damage, since neutrophils influence the activation state of the subsequent immune cell populations (15). Different immune cells sequentially arrive to the site of the injury and this inflammatory response is orchestrated by the neutrophils, as discussed below.

Neutrophils produce a variety of growth factors like fibroblast growth factor (FGF), hepatocyte growth factor (HGF), insulin-like growth factor-1 (IGF-1), vascular endothelial growth factor (VEGF), transforming growth factor (TGF)-β1 and inflammatory cytokines and chemokines including TNF-α, IL-6, CCL17 and CCL2. This last chemokine is the main responsible for the attraction of monocytes into the site of injury (5). During this first phase, there is a predominance of the secretion of proinflammatory factors like TNF-α, interferon (IFN)-γ, IL-1β, IL-2, IL-6, CXCL1, CXCL4, CD40L and stromal cell-derived factor (SDF) (14). When monocytes enter the muscle, they get exposed to IFN-γ and TNF-α and differentiate into macrophages (15). This Th1 cytokine profile polarizes macrophages into the pro-inflammatory type 1 (M1) (15). M1 macrophages along with neutrophils can adapt to a low pH environment and are responsible for the phagocytosis of debris (14). Also, M1 macrophages contribute to cell lysis and regulate the proliferation and differentiation of myoblasts. Initially, M1 macrophages promote the proliferation and inhibit the differentiation of MPC through the secretion of IGF, TGF-β, FGF, platelet-derived growth factor (PDGFBB), and epidermal growth factor (EGF). Also, they produce TNF-α, nitric oxide (NO), IL-12, IL-1, and IL-6, being this last cytokine a key factor for the induction of the proliferation of MPC (14). At the same time, the high concentrations of IFN-γ inhibit the differentiation of myoblasts by decreasing the expression of myogenin (15). Therefore, the immune system is also responsible for the activation of MPC and the regulation of their differentiation into myotubes (13).

## Regeneration phase of the muscle repair

Between 4-7 days after muscle injury, there is a transition from a pro-inflammatory to an anti-inflammatory environment with an M2 macrophage phenotype predominance. In this second phase, there is an increased secretion of anti-inflammatory and pro-fibrotic molecules like IL-27, HGF, TGF-β, IL-10 and IL-22 (14). Annexin A1, secretory leukocyte protease inhibitor (SLPI), glucocorticoid leucine zipper (GILZ), and developmental endothelial locus (DEL)-1 are the mediators that induce the transformation of macrophages from a pro-inflammatory to an anti-inflammatory phenotype (Reviewed in 18). Although the two types of macrophages are thought to appear sequentially in muscle injuries, the dichotomy between M1 and M2 macrophages is not a reflection of the plasticity of these cells. Some of them can express biomarkers of both populations at the same time and there can be macrophages with both phenotypes at any stage of the muscle regeneration (15).

## Muscle remodeling after injury

**Figure 1:**
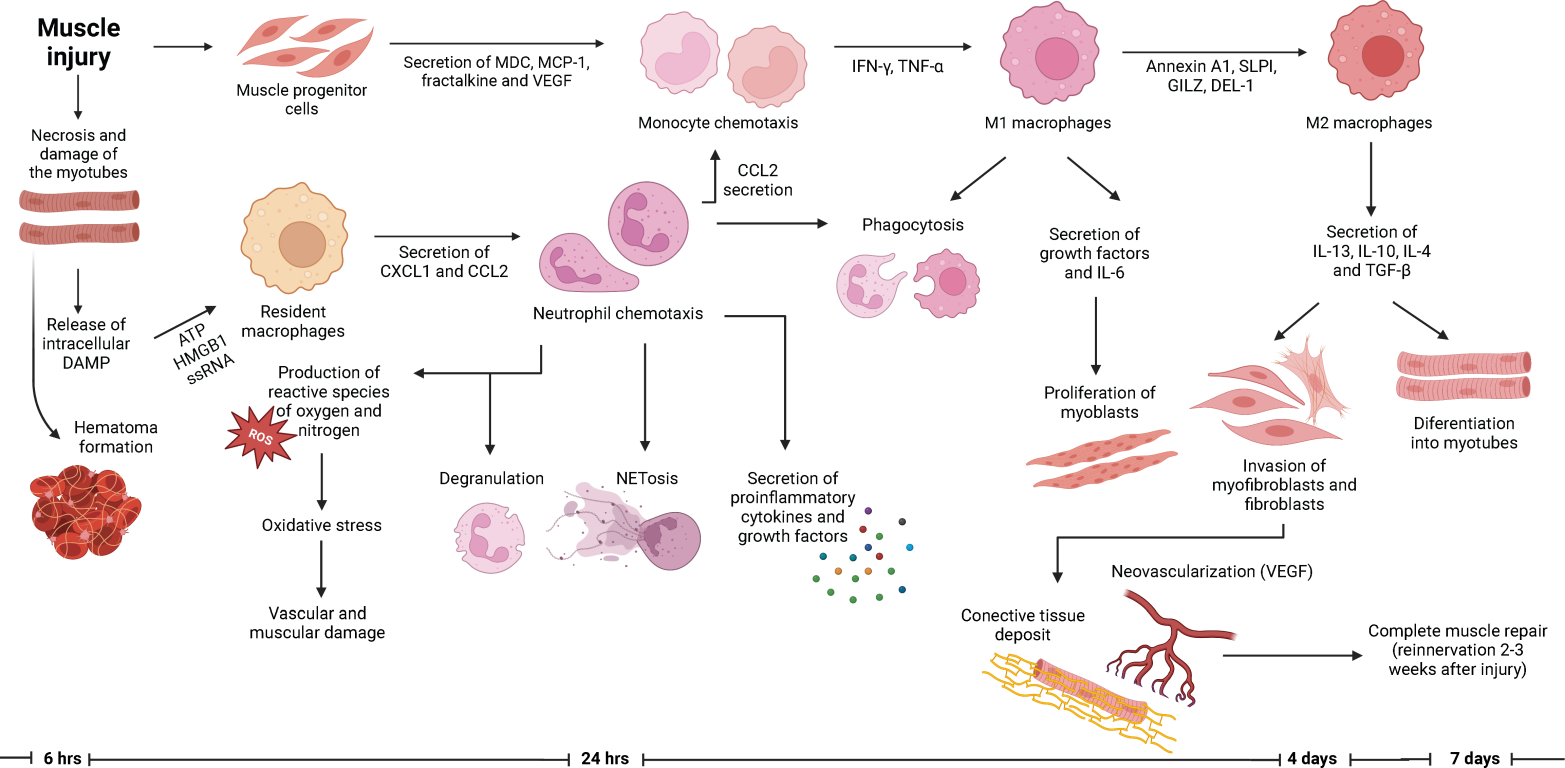
Role of neutrophils in the muscle regeneration process. Muscle injury encompasses the necrosis and degeneration of myofibers with hematoma formation. The arrival of neutrophils into the site of injury occurs within hours after the lesion. Neutrophils are responsible for orchestration of the subsequent inflammatory response, which eventually will lead to a successful muscle repair. Along with neutrophils, macrophages are responsible for the phagocytosis of debris and the regulation of the proliferation and differentiation of myoblasts. Specifically, M2 macrophages promote the differentiation of myoblasts into myotubes and the production of connective tissue which will serve as a scaffold for the new myotubes. Afterwards, the muscle repair is completed with the neovascularization and reinnervation of the previously damage tissue, which allows the recovery of the contractile forces.

In this phase of muscle repair, M2 macrophages secrete IL-13, IL-10, IL-4 and TGF-β, inhibit myoblast proliferation and promote their differentiation into new myotubes or their fusion with previously damaged myofibers. They also induce the growth of myofibers and promote angiogenesis and the production of extracellular matrix (14). During this remodeling process, the previously deposited fibrin and fibronectin create a matrix that is invaded by fibroblasts. The secretion of TGF-β1 participates in the proliferation of fibroblasts and myofibroblasts and promotes the production of type I/III collagen, laminin and fibronectin, which generates a scaffold for the new myofibers (5). The synthesis of connective tissue is mediated by the connective tissue growth factor, platelet derived growth factor and myostatin. Also, the neovascularization occurs during this phase through the secretion of VEGF (5). The last part of the muscle repair is the reinnervation, which occurs 2-3 weeks after injury (5). Once the muscle repair is completed, macrophages return to their baseline status probably by migrating through the lymphatic vessels (Reviewed in 19). In **Figure 1**, we summarize the muscle regeneration process, with emphasis on the function of neutrophils.

As discussed above, the main function of neutrophils after a muscle injury is the orchestration of the initial inflammatory response. The types of inflammatory cells that infiltrate the muscle influence the severity of the injury and the regeneration process (20). Among the different types of immune cells, neutrophils possess the greater capacity to induce muscle damage, since they synthetize reactive oxygen species and nitrogen and induce myotubular cell membrane damage (20). The excessive muscle infiltration by neutrophils because of an augmented secretion of TNF-α, IL-6, CCL17 and CCL2, can hamper the regeneration capacity of the muscle and may even magnify the initial muscle lesion as we will describe in the following section.

## Muscle damage induced by the excessive effector functions ---of neutrophils

The pathogenic effects of neutrophils on the skeletal muscle have been demonstrated in animal models of muscle injury. For instance, as reviewed by Pizza et al. in 2005, in animal models of Duchene’s muscle dystrophy, and of lengthening contractions-induced muscle injury (21), neutrophils are recruited within minutes and release MPO, which catalyzes the production of hypochlorous acid, hydrogen peroxide and chloride, increasing the oxidative stress (11). Also, in animal models of mechanical loading after 10 days of hindlimb immobilization, there is lysis of the cell membrane with necrosis of myofibers (22). Once the mobility of a previously immobile muscle is restored, the movement increases the loading stress and the MPO activity in neutrophils, which invade the muscle within 2 hours of physical activity (22). In this model of muscle injury, when myocytes previously exposed to mechanical loading were co-cultured with mouse peritoneal neutrophils, there is myocyte destruction even in the absence of MPO, implying that some of the neutrophil-mediated mechanisms of myocyte destruction may be independent of this enzyme (22). The addition of superoxide dismutase (SOD) further increased the lysis of myocytes, probably related to the enhanced production of hydrogen peroxide induced by this enzyme, which is afterwards converted to hypochlorous acid (22). The pathogenic effect of neutrophils and MPO in this animal model was confirmed since the muscle cell lysis was reduced in MPO knockout (KO) mice (22).

The deleterious effect of excessive neutrophil activation has also been explored in animal models of volumetric muscle loss, in which the exposure of MPC to the secretory products of neutrophils resulted in an inhibition of the fusion of C2C12 cells (23). *In vivo*, the effect of the modulation of the function of neutrophils on muscle regeneration was tested in an animal model of snake venom toxin (notexin)-induced muscle injury combined with hindlimb ischemia (24). This is a model of severe and prolonged muscle injury with direct damage to muscle fibers without any effect on MPC, connective tissue or immune cells (24). Seo et al, evaluated the application of mechanotherapy in the form mechanical loading in this model of muscle injury as a muscle-regeneration strategy (24). Initially, the authors created a robotic device integrating an electromagnetic actuator and a force sensor assembly covered with silicone. The mouse limb was restrained in the desired position in a silicone-based mount. The device allowed the generation of mechanical loads of arbitrary magnitude, frequency, duty cycle and shape. Also, the tissue strain in the stimulated region of the muscle was visualized with a high frequency ultrasound transducer (24). One day after the ischemic surgery, mice with the more severe injuries were equally selected to receive either no treatment or mechanical loading. The treatment was applied from the first day of the muscle lesion and up to 14 days. The histological and functional analyzes were performed on day 14 (24).

The mechanical load significantly reduced the fibrous tissue production, the percentage of damaged muscle fibers and was related to an increased in muscle fibers with a higher cross-sectional area and a progressive increased in the tetanic force, reaching its maximum on day 14 (24).

The mechanical loading treatment induced changes in the tissue recovery process. As evaluated by real time quantitative polymerase chain reaction, the treatment promoted a higher expression of the adult myosin heavy chain 4 and a decrement in the expression of the embryonic (Myh3) and perinatal (Myh8) myosin heavy chains, which are mainly produced during muscle injury. There was not an effect of the mechanical loading treatment on the perfusion of the damaged muscle as compared with the non-damaged hindlimb (24).

Afterwards, the authors assessed if the mechanical load treatment was related to a differential expression of cytokines, chemokines, and growth factors. To this end, they evaluated 111 inflammatory markers in mice. The main changes in the inflammatory cytokines and chemokines were found at day 3 after injury. The inflammatory markers that were decreased at least 50% were related to myeloid linage, specifically to neutrophil chemotaxis and migration. Also, Ly6G proinflammatory neutrophils were diminished at day 3 after muscle injury in mice treated with mechanical loading (24)..

To assess the effect of neutrophils on muscle regeneration *in vitro*, the authors isolated neutrophils from C57BL6/J mice after GM-CSF treatment. Afterwards, they activated the neutrophils with PMA and isolated the conditioned media containing neutrophil derived factors. The authors incubated the myoblasts with the neutrophil conditioned media. This experiment resulted in an increased proliferation but a diminished differentiation of MPC (24). The authors evaluated the relative expression of the inflammatory markers in the neutrophil conditioned media in comparison with the basal media which was Roswell Park Memorial Institute (RPMI) without neutrophils and the relative expression of these markers in the muscle from mice treated with the mechanical loading in comparison with the injured muscle without treatment. Four key factors were found to be overexpressed which were CCL3, MMP-9, LCN2 (lipocalin 2) and CXCL2. Neutralization of LCN2 and CXCL2 had moderate to no impact on MPC proliferation (despite its previous associations to disease activity in SLE and juvenile idiopathic arthritis), however CCL3 and MMP-9 were proved to influence the proliferation and differentiation of MPC. Antibodies blocking CCL3 and MMP-9 inhibited the MPC proliferation, whilst antibodies against MMP-9 favored the differentiation of MPC into myotubes (24).

To confirm the role of neutrophils in this model, the authors depleted the neutrophils in mice after the muscle injury using an anti-Ly6G antibody and compared the results with the mechanical load treatment. At day 14, the depletion of neutrophils reduced the percentage of damaged muscle fibers and fibrosis. Also, neutrophil depletion led to an increased amount of large muscle fibers and a greater recovery of the contractile force (24). These results emphasize the importance of an adequately modulated initial inflammatory response of neutrophils for the successful muscle regeneration.

## Potential pathogenic role of the NETosis on muscle repair after injury

As part of the effector functions of neutrophils, the pathogenic effects of NETs after muscle injury have also been demonstrated. In an animal model of IR-induced muscle injury, the accumulation of structures containing DNA, MPO and citrullinated histone 3 (cH3) (NETs) was observed within 1-3 days after injury (12). Nonetheless, the inhibition of NETosis using Cl-amidine and by silencing the PAD4 gene did not influence muscle regeneration or fibrosis (12). In an animal model of muscle injury induced by intramuscular injection of monosodium urate, Suzuki et al. observed the deposit of NETs and the infiltration of neutrophils. This phenomenon was related to a decreased mechanical nociceptive threshold (25). At the same time, the excessive muscle contraction induced by repetitive electrical stimulation also promoted a decreased mechanical nociceptive threshold and the production of NETs (25). The muscle hyperalgesia was reversed using intravenous DNase I and neutrophil depletion (25), which supports a potential role of NETs in the hyperalgesia after muscle injury. NETs also have a role in sterile muscle injury. In 2019, Liu et al. found that after acute peripheral tissue trauma, endogenous mitochondrial DNA (mtDNA) and oxidized mtDNA was released, which induce the formation of NETs (26). Oxidized mtDNA was especially effective for the induction of NETosis, through the activation of neutrophils by the cyclic guanosine monophosphate (GMP)-adenosine monophosphate (AMP) synthase (cGAS)-STING and TLR9 pathways. mtDNA also increased the production of neutrophil elastase, ROS, and PAD4 leading to enhanced NETosis during this process and highlighting the association between NET formation and sterile inflammation, probably through the stimulation of pattern-recognition receptors (26). These data suggest that sterile inflammation after muscle injury induces the release of NETs, which may be related to the presence of muscle pain but does not influence its regeneration.

In summary, neutrophils have a dual role on muscle regeneration after injury. It is clear that neutrophils are fundamental for the orchestration of the inflammatory response that will eventually lead to muscle repair. Nonetheless, an excessive effector function of neutrophils induces tissue damage and hampers the muscle regeneration capacity. The threshold for the overactivation of neutrophils after muscle injury is unknown, but apparently depends on the severity of the muscle necrosis with the consequent release of DAMP during the initial lesion, as shown in models of IR injuries (12). This hypothesis is supported by the study of Larouche JA et al, showing that volumetric muscle loss is related to an intense infiltration of natural killer cells and the persistence of neutrophils for over two weeks, hampering the muscle regeneration and leading to fibrosis (23). Besides their role on muscle generation, neutrophils are very important in the pathogenesis of chronic muscle injuries, like those observed in IIM as discussed in the following section.

## IIM as systemic autoimmune diseases

IIM are a group of heterogeneous autoimmune diseases characterized by myositis and variable extramuscular features (Reviewed in 27). Different clinical phenotypes are included in this spectrum of diseases, which are the juvenile and adult dermatomyositis (JDM and DM respectively), polymyositis (PM), anti-synthetase syndrome (AS), immune mediated necrotizing myopathies (IMNM) and overlap syndromes (27). Although IIM are rare diseases, they are an important cause of morbidity and premature mortality due to infections, cancer, cardiovascular and interstitial lung diseases (Reviewed in 28). In IIM, the clinical features are closely associated to the presence of the myositis specific and associated antibodies (MSA and MAA respectively) (29–32), which are present in approximately 70% of patients (Reviewed in 33). The autoantibodies associated with DM are the anti Mi-2, transcription intermediary factor (TIF)1-γ, nuclear matrix protein (NXP)-2, small ubiquitin-like modifier (SUMO)-1 and melanoma differentiation-associated protein (MDA) 5. The anti-synthetase syndromes are related with the positivity of anti-aminoacyl tRNA synthetases antibodies, being the most frequent the anti-histidyl synthetase antibody (Jo1). The characteristic autoantibodies of the IMNM are the anti-3-hydroxy 3-methyl-glutaryl coenzyme A reductase (HMGCR) and anti-signal recognition particle (SRP) (Reviewed in 34).

Although the immunopathogenesis of IIM is not completely understood, previous studies have shown the overexpression of proinflammatory cytokines like IL-1α, IL-1β, TNF-α (Reviewed in 35), IL-18 and type I and II IFN (36) in peripheral blood and muscle of patients with this group of diseases. Most of these mediators are chemotactic for neutrophils, probably explaining that neutrophilia is a biomarker of disease activity in IIM, as discussed below. Besides the activation of the innate and adaptive immune response, certain types of IIM like DM and AS are characterized by other pathogenic features like vasculopathy. In IIM, the vascular damage is characterized by ectasia, endothelial damage and fibrin deposits and is more prominent in the skin of patients with DM in comparison with other autoimmune diseases like SLE (37). In muscle, there are ultrastructural changes like swollen endothelial cells, margination of leukocytes and micro-thrombi of platelets (38). The muscle biopsy findings of patients with anti Jo1 antibodies include perifascicular necrosis and a vasculopathy similar to DM (39). The vasculopathy of patients with IIM is associated with an increased production of reactive species of oxygen and nitrogen (40) which highlights the potential role of neutrophils in this complication.

In the following sections, we will initially discuss the role of neutrophils as biomarkers of disease activity in IIM. Also, we will address the potential role of neutrophils and NETs in the pathogenesis and tissue damage induction in this group of diseases.

## Normal density (conventional) neutrophils and NETs as biomarkers of disease activity in IIM

In peripheral blood, neutrophils are the most abundant leukocytes and have been evaluated as biomarkers of different clinical features in patients with IIM. Previous studies have demonstrated that the neutrophil-lymphocyte ratio (NLR) is correlated with certain disease activity indexes like the myositis disease activity assessment (MYOACT) (coefficient=0.43) (41). The role of neutrophils and their products has been extensively studied in the context of IIM-associated interstitial lung disease (ILD). In patients with clinically amyopathic dermatomyositis (CADM), there was an overexpression of IL-8 in neutrophils, especially during periods of active disease and this cytokine is significantly associated with anti-MDA5-ILD (42). In another retrospective study that included patients with CADM, DM and PM, it was found that subjects who died due to causes related to the disease like ILD had a higher NLR at baseline. After a multivariate analysis, the NLR was an independent risk factor for death in patients with IIM (hazard ratio (HR) 2.216 95% confidence interval (CI) 1.923-14.06), P=0.001) (43). In patients with MDA5+ DM, a NLR of less than 4.86 has been found to be related to an improved overall survival with a sensitivity of 0.74, specificity of 0.62 and positive and negative predictive values of 0.43 and 0.85 respectively (44). In another study of 156 patients with IIM, 36 of them had ILD. The 9 patients who presented a torpid course, characterized by chronic respiratory failure, treatment refractoriness and death showed a neutrophilic bronchoalveolar lavage (45).

On the other hand, NE was found to be elevated in peripheral blood of patients with IIM, especially in those with DM in a cross-sectional study (46). The amount of NE was higher in patients with active myositis, showing an area under the curve (AUC) of 0.9 for IIM, 0.9 for DM and 0.88 for PM (46). The NE levels correlated with the MYOACT r=0.549 (P<0.001) in IIM, r=0.543 (P<0.001) in DM and r=0.506 (P<0.05) in PM (46). Also, the elastase to neutrophil ratio (ENR) was higher in both patients with active DM and PM with an AUC of 0.96 for IIM, 0.96 for DM and 1 for PM (46). Besides, there was a correlation between the ENR and the MYOACT in IIM with an r=0.655 (P<0.0001), r=0.672 (P<0.001) in DM, and r=0.661 (P<0.01) in PM (46). Furthermore, there was a positive correlation between the serum NE, the ENR and the muscle enzymes, c-reactive protein, and erythrocyte sedimentation rate (46). Patients with anti PM-Scl antibodies had a higher ENR (46). In another study of patients with IIM, it was found that the serum levels of IL-6, NE and MPO-DNA complexes were higher in subjects with respiratory infections and ILD (Reviewed in 47). The AUC for the prediction of respiratory infections of these biomarkers were 0.692 (95% CI 0.532-0.861, P=0.044) for MPO-DNA complexes, 0.692 (95% CI 0.5251-0.859, P=0.044 for NE and 0.72 (95% CI 0.561.0.880, P=0.021) for IL-6 (47).

Neutrophilia has also been reported as a predictor of other clinical features in patients with IIM (48). In a study of patients with anti-TIF1γ+ DM, it was observed that a higher amount of neutrophils and an increased NLR were biomarkers of patients at high risk of cancer development (48).

Regarding the NETs, the identification of remnants of these structures measured as the NE-DNA and MPO-DNA complexes is a standard way to detect circulating NETs in serum from patients with autoimmune diseases (Reviewed in 49). NETs remnants are observed in peripheral blood of patients with IIM, which suggests that the serum or plasma from patients with these diseases may promote the spontaneous release of NETs. In this regard, Peng et al. decided to evaluate if the serum from patients with anti-MDA5 antibodies was able to promote NETosis *in vitro* (50). In this study, an enhanced production of NETs was observed after the stimulation of neutrophils with sera from anti-MDA5+ DM patients. The authors also found that the amount of circulating free (cf) DNA and LL-37 was higher in patients with anti MDA5+ rapidly progressive ILD (50). Nonetheless, it is important to acknowledge the limitations of these findings, since the sole detection of cfDNA is not an accurate indicator of the presence of NETs. Other types of cell death including apoptosis, necrosis, and pyroptosis are a source of cfDNA (51). Furthermore, cfDNA can be actively secreted by different cells (Reviewed in 51). Therefore, further methods were required to confirm the presence of circulating NETs and their content of LL-37 in patients with IIM. To this end, our group confirmed that neutrophils from patients with active DM produce NETs containing LL-37 using immunofluorescence and confocal microscopy (52). According to these previous reports, in the paper by Seto et al, the circulating NETs remnants measured as the NE-DNA and MPO-DNA complexes were increased in patients with DM and JDM and in those with positive anti-MDA5 and anti-TIF1-γ antibodies (10). The authors also analyzed whether the purified anti-MDA5 antibodies from patients with DM were able to induce NETosis *in vitro*. They found that the anti-MDA5 antibodies promoted the production of NETs (10). Although the association between NETs and the clinical features of IIM results exciting, it is important to acknowledge that the method to measure the MPO-DNA complexes by ELISA has limitations. In fact, recent studies have shown that the detection of MPO-DNA complexes by this method does not accurately reflect the amount of NETs (53) and therefore, more precise techniques should be developed to confirm the presence of NETs *in vivo.*

The invasion of neutrophils and the NETs’ deposit have been documented in different tissues of patients with IIM. In skin lesions from patients with DM, the neutrophils (MPO+) are the second most abundant cells and can be found in the perivascular space of the upper dermis and epidermis (54). Previous studies have demonstrated the presence of neutrophils expressing LL-37 in skin and muscle biopsies of patients with IIM (55). The presence of neutrophils and NETs in skin, muscle and lung biopsies of patients with IIM was confirmed by Seto et al. (10). Supporting the role of neutrophils in the development of calcinosis, in patients with JDM it was found that neutrophils infiltrate the muscle, are adjacent to the areas of calcium deposit and contain phagocytized calcium crystals (56). The phagocytosis of calcium crystals activated the neutrophils and induced the production of NETs containing mitochondrial DNA (56). In JDM patients with anti-TIF1-γ and anti-MDA5 antibodies, a decreased capacity for the degradation of NETs was observed and the NETs remnants in peripheral blood correlated with the serum levels of IL-8 and immune complexes (56). From this study, it is not clear whether the release of NETs after stimulation with calcium crystals is a normal response from these cells or the NETs contribute to the development of calcinosis. We hypothesize that neutrophils and NETs induce vascular damage in IIM, leading to ischemic dystrophic calcifications of the skin, but this theory requires further confirmation. Our group also report a relationship between the circulating NETs remnants and calcinosis in adult patients with IIM (52). In the mentioned study, the amount of NETs was also higher in patients with anti-PMScl. The involvement of NETs in the PM-Scl phenotype is interesting because those patients are characterized by the presence of systemic-sclerosis overlap features with Raynaud’s phenomenon, calcinosis and ILD and pulmonary hypertension (57).

Currently, it is not clear whether NETs have a pathogenic effect on mature muscle cells. To assess this question, Seto et al. differentiated human skeletal muscle myoblasts into myotubes *in vitro* and co-cultured them with NETs. A decreased viability of myotubes was observed and this mechanism was dependent on the presence of histone (H) 4 since the pathogenic effect of NETs in myotubes was partially reversed using an anti-H4 antibody (10) nonetheless, this experiment was limited because the authors only evaluated the size of the myotubes after their exposure to NETs measuring the area of the myocytes that were positive for the myosin heavy chain without assessing viability or differentiation markers. Also, it is unknown if the NETs from patients with IIM induce any kind of pathogenic effect on myotubes, which warrants further studies.

NETs may promote the progression of ILD in patients with IIM. In an animal model of polymyositis induced by allogenic injection of rat muscle emulsion, mice developed lung inflammation and fibrosis. Nonetheless, after the intraperitoneal administration of NETs, the pulmonary fibrosis was enhanced and mice showed a higher expression of MPO and α-smooth muscle actin (SMA) as markers of NETosis and of myofibroblasts’ differentiation respectively (58). Also, there was an upregulation of TLR9 and Smad2 (58). *In vitro*, the stimulation of lung fibroblasts with PMA-induced NETs favored their proliferation and the expression of genes related to fibrosis like ACTA2, CCN2 and ADAM12, which was reversed after treatment with DNase and inhibitors of MPO and H3 (58). This mechanism was dependent on TLR9 signaling (58). Until now, most studies addressing the release of NETs in IIM have been focused on suicidal NETosis, as this is the paradigm in most autoimmune diseases. Nonetheless, in the study by Duvvuri B et al. (56), the release of NETs with mitochondrial DNA was observed. Besides, since one of the main stimuli for the production of NETs in sterile muscle inflammation is the activation of TLR, the complement activation is a fundamental pathogenic mechanism in IIM, the currently unexplored role of the vital release of NETs should be assessed in this group of diseases. This may be of special interest in DM, since NETs containing mitochondrial DNA are especially interferogenic (59).

Patients with IIM are not only characterized by an enhanced production but also present a lower capacity to eliminate NETs (60). In a study by Zhang et al. that included 72 adult Asian patients with DM, 25 patients with PM and 54 healthy donors it was observed that conventional neutrophils from these patients undergo spontaneous NETosis (60). Also, when neutrophils were exposed to plasma from patients with IIM, the production of NETs was enhanced (60). Furthermore, in patients with ILD, a decreased plasmatic degradation capacity of NETs was detected, which was attributed to a low DNase I activity (60). Interestingly, the plasmatic low DNase activity was more prominent in anti Jo1+ patients and was improved after treatment with glucocorticoids (60). These studies confirm the role of the expansion of neutrophils in peripheral blood as biomarkers of distinctive clinical features of patients with IIM. Also, they portray evidence of the invasion of neutrophils and the production of NETs in skin, lung, and muscle of patients with IIM.

## The role of low-density granulocytes and in the pathogenesis of IIM

Low density granulocytes (LDG) are neutrophils isolated along with peripheral blood mononuclear cells (PBMC) after the separation of blood by density gradients with centrifugation (Reviewed in 61). These cells are characterized by a decreased phagocytic capacity, but an enhanced production of NETs (61) and have been shown to be expanded in many autoimmune and autoinflammatory diseases (Reviewed in 62).

The first description of the expansion of LDG in IIM was made by Zhang et al. in patients with DM (63). Their study included 48 adult Asian patients with DM and 19 healthy donors. Twenty-eight patients had ILD at recruitment. In comparison to healthy donors, patients with DM had an expansion of LDG in peripheral blood (63). The proportion of LDG was 2.7 times higher in patients with ILD, and this increment was more pronounced in patients with subacute pneumopathy (63). The amount of LDG correlated with the circulating free cfDNA and LL-37, suggesting that LDG are a potential source of NETs in patients with DM (63). Interestingly, the authors observed an increased production of LL-37 in PBMC of patients with DM, which further supports the hypothesis that in this disease, LDG may produce NETs enriched with this antimicrobial protein (63). The expansion of LDG in both children and adult patients with IIM was confirmed by Seto, et al. LDG from these patients were characterized by an enhanced production of NETs (10).

LDG also contribute to the appearance of other clinical features in patients with IIM. Our group described that CD10+ LDG were expanded in peripheral blood of patients with active IIM (52). The proportion of LDG correlated with the muscle enzymes, the patient’s visual analogue scale of disease activity, the myositis intention to treat index (MITAX), MYOACT and manual muscle test (MMT) 8 (52). Furthermore, the proportion of LDG correlated with the muscular and gastrointestinal damage accrual as well as with the damage severity and extension and the disability measured with the health assessment questionnaire (HAQ) (52). The CD10+ LDG correlated with the serum levels of IL-18, which is a key cytokine involved in the disease activity of patients with IIM (52). In accordance with the previously reported association between the presence of vascular damage and LDG, we found that patients with an abnormal nailfold capillaroscopy had a higher proportion of these cells. CD10+ LDG were elevated in patients with dysphagia and dermatosis (52). the proportion of LDG was higher in patients with anti-MDA5 antibodies. The contribution of LDG to the clinical phenotype of patients with anti-MDA5 antibodies is especially interesting, since the MDA5 phenotype is characterized by rapidly progressive ILD (64) and intense vasculopathy manifested as cutaneous ulcers and necrosis (65).

In regard to the expansion of LDG in peripheral blood of patients with IIM, it is worth noting that not all LDG have a pro-inflammatory phenotype. Our group identified the presence of LDG with a phenotype corresponding to myeloid-derived suppressor cells (MDSC) in patients with IIM, especially those with active disease (66). These polymorphonuclear (PMN)-MDSC were characterized by the expression of PD-L1 and Arginase-1. The expression of PD-L1 in PMN-MDSC correlated with parameters of disease activity and damage accrual and with proinflammatory serum cytokines like IL-6, IL-8 and GM-CSF (66). Future studies about the regulatory functions and significance of these cells in IIM are necessary.

In **Table 1** we summarize the clinical phenotypes of the IIM and their relationship with distinctive types of neutrophils.

**Table 1:** IIM subgroups, their main associated autoantibodies, clinical manifestations, neutrophil biomarkers and LDG phenotypes.

IIM	MSA/MAA	Clinical features	Neutrophil biomarkers	Associated LDG phenotype
JDM DM CADM	Mi-2 TIF-1γ NXP2 SUMO-1 MDA5	Cutaneous rash Vasculopathy ILD Dysphagia Myositis Calcinosis	NETs (MPO-DNA complexes) IL-8 NLR ENR	LDG CD10+ LDG CD10- PMN-MDSC
PM	None	Myositis	NLR ENR	LDG CD10+ LDG CD10-
AS	Jo-1 PL7 PL12 OJ EJ KS Zo SC JS YRS	Fever Rash ILD Myositis Arthritis Mechanics hands Raynaud phenomenon Myocarditis	NETs (MPO-DNA complexes) NLR ENR	LDG CD10+
IMNM	HMGCR SRP	Dysphagia Cardiac involvement (anti-SRP) Necrotizing myopathy	Not determined	Not determined
Overlap syndromes	U1RNP Ku PM- Scl	Myositis Head drop Muscle weakness Necrotizing myopathy Mechanics hands	NETs (MPO-DNA complexes)	Not determined

IIM, Idiopathic inflammatory myopathy; MSA, Myositis specific antibodies; MAA, Myositis associated antibodies; LDG, Low density granulocytes; JDM, Juvenile dermatomyositis; DM, Dermatomyositis; CADM, Clinically amyopathic dermatomyositis; ILD, Interstitial lung disease; NLR, Neutrophil to lymphocyte ratio; ENR, Elastase to neutrophil ratio; HMGCR, 3-hydroxy 3-methylglutaryl coenzyme A reductase; SRP, Signal recognition particle.

## NETs as potential therapeutic targets in muscle injuries and IIM

The modulation of NETosis as a therapeutic approach in any autoimmune disease is mainly based on the prevention of their release or the enhancement of the degradation of the accumulated NETs (7). The evidence assessing the targeting of NETosis as a treatment of muscle injury has been controversial. In one study, the KO of TLR4 led to a decrement in the amount of NETs and the severity of IR injury in a murine model (67). Unfortunately, the sole modulation of NETs was not beneficial for the treatment of IR injuries in subsequent experiments (68). Conversely, the reduction of NETs after the administration of batroxobin, an inducer of defibrinogenation improved the microcirculation and the outcome of ischemic muscle injury (69). These experimental data suggest that not only NETosis but all the effector mechanisms of neutrophils are fundamental inducers of muscle damage and may represent therapeutic targets.

Although it is unknown if the drugs used to treat the clinical features of IIM exert their role through inhibition of NETosis, some of them have demonstrated benefits in other autoimmune and autoinflammatory diseases, in which NETosis is a key pathogenic element. For instance, hydroxychloroquine is frequently prescribed to treat the cutaneous features of DM patients and it has been shown to decrease the release of NETs by the inhibition of the TLR9 stimulation (Reviewed in 70). Other inhibitors of the production of ROS such as methotrexate and prednisone may indirectly reduce the production of NETs (70). Also, since NETosis is promoted by immune complexes in patients with JDM, the depletion of B cells using rituximab or the reduction of auto-antibodies using intravenous immunoglobulin (IVIG) may also be able to decrease the amount of auto-antibodies and indirectly, the release of NETs (70, 71). The type I IFN blockers may also have a role in the inhibition of NETosis in IIM as has been shown in patients with SLE (Reviewed in 71), but this hypothesis warrants further testing. The degradation of the excessively produced NETs using recombinant human DNase has shown some beneficial effects in animal models of SLE and anti-phospholipid syndrome (71) and may be a potential therapeutic intervention in patients with IIM.

**Figure 2:**
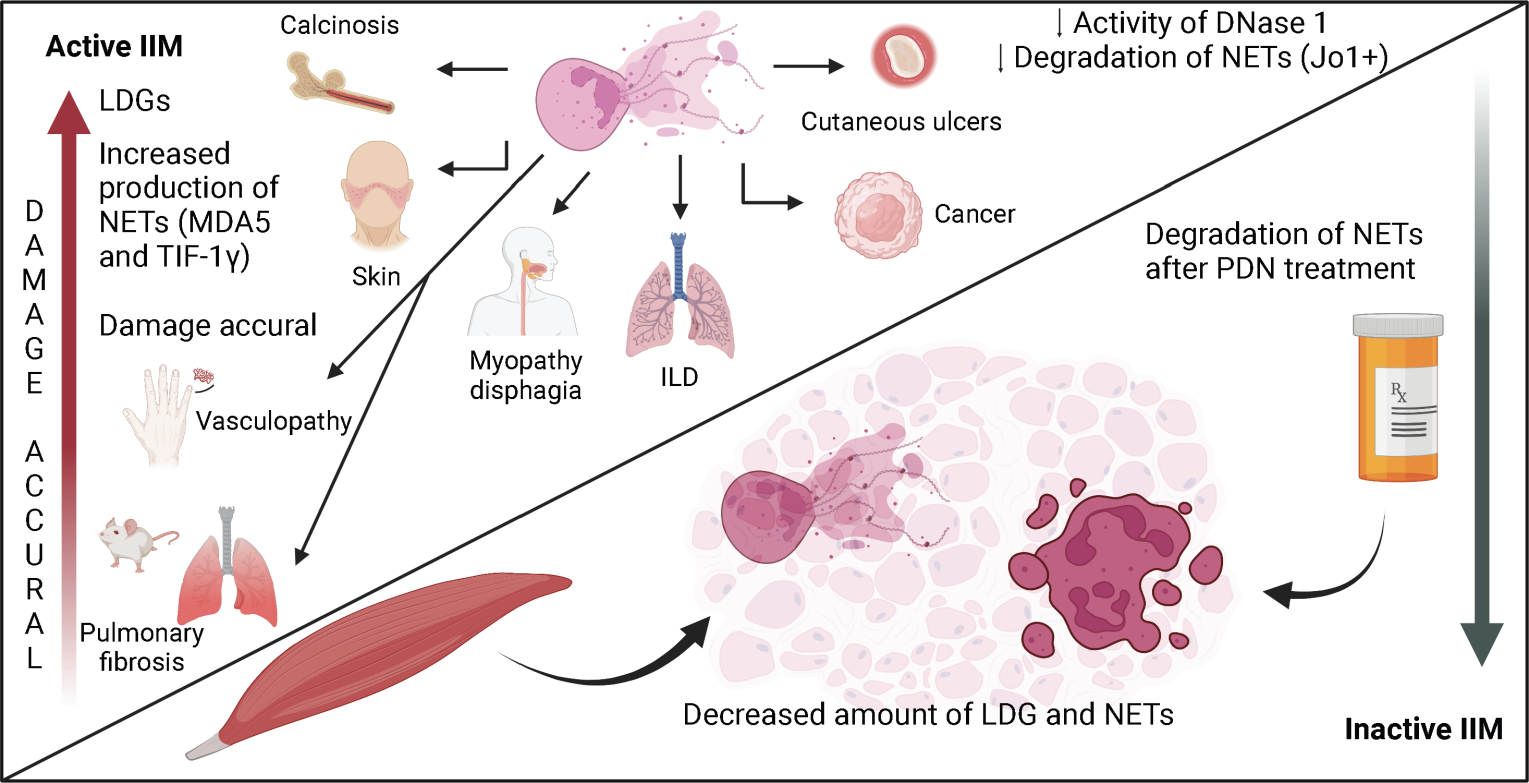
The role of neutrophils and NETs in the immunopathogenesis of IIM. The expansion of neutrophils in peripheral blood is a biomarker of disease activity and a sign of bad prognosis in patients with IIM. Neutrophils have been related to the presence of ILD and cancer. In patients with active disease, there is an expansion of LDG, which are subset of neutrophils with enhanced production of NETs. In patients with inactive disease under prednisone treatment, the amount of LDG decreases. The expansion of these cells in peripheral blood has been related to certain clinical features like dermatosis, calcinosis, cutaneous ulcers, vasculopathy and dysphagia. NETs are found in peripheral blood and in skin, muscle and lung biopsies from patients with IIM. During active disease, there is enhanced production and deficient degradation of NETs, especially in patients with dermatomyositis and Jo1+ anti-synthetase syndrome. The impaired degradation capacity of NETs is due to a decreased plasmatic DNase-1 activity, which is partially reversed after treatment with glucocorticoids.

In **Figure 2**, we summarize the role of LDG and NETs in the immunopathogenesis of IIM, and the effect of glucocorticoid treatment on the disease activity, the proportion of LDG and the degradation of NETs.

## Conclusions

Neutrophils are fundamental orchestrators of the initial immune response that will eventually lead to a successful muscle generation after an injury. Nonetheless, a dysregulation of the effector functions of neutrophils during the repair of a muscle injury may indeed expand the damage and cause irreversible disability. In chronic muscle diseases like IIM, neutrophils are prognostic and clinical markers of disease activity and are related to the presence of ILD and cancer. An expansion of a special subset of neutrophil called LDG is found in patients with IIM. These cells may be proinflammatory or have a phenotype corresponding to MDSC. In IIM, LDG correlate with parameters of disease activity and damage as well as with pro-inflammatory serum cytokines. Also, LDG are expanded in patients with IIM and ILD, dermatosis, vasculopathy, and dysphagia. LDG are characterized by an enhanced production of NETs, which have been involved in the pathogenesis of skin, lung and muscle disease in patients with IIM. The NETs have an important role in the development of calcinosis. During episodes of disease activity, patients with IIM present an enhanced production and low degradation capacity of the NETs. The low degradation capacity of the NETs is due to a decreased DNase-1 activity, which is partially reversed after treatment with glucocorticoids. The accumulation of these structures may induce tissue damage and promote lung fibrosis as has been shown in animal models of PM.

## Author contributions

JT-R, BA-C, RA-A and DG-M participated in the conceptualization of the review and wrote the original draft. All authors contributed to the article and approved the submitted version.
